# Autologous Vas Deferens Sling for Early Urinary Continence During Retzius-Sparing Robot-Assisted Radical Prostatectomy: A Randomized Controlled Clinical Trial

**DOI:** 10.3390/healthcare14142090

**Published:** 2026-07-13

**Authors:** Zhiyuan Yang, Jiyuan Sun, Jingxian Xu, Haifeng Huang, Fan Zhang, Shengjie Zhang, Wei Chen, Xuefeng Qiu, Junlong Zhuang, Linfeng Xu, Hongqian Guo, Qing Zhang

**Affiliations:** 1 Department of Urology, Nanjing Drum Tower Hospital, The Affiliated Hospital of Nanjing University Medical School, Nanjing 210008, China; 522023350121@smail.nju.edu.cn (Z.Y.); 522023350183@samil.nju.edu.cn (J.S.); 522023350112@smail.nju.edu.cn (J.X.); dr.dahuangfeng@aliyun.com (H.H.); zhangfan20082002@aliyun.com (F.Z.); 15250954937@163.com (S.Z.); zyzxcw@126.com (W.C.); xuefeng_qiu@nju.edu.cn (X.Q.); zhuangjl@nju.edu.cn (J.Z.); linfengxu1107@hotmail.com (L.X.); 2 Institute of Urology, Nanjing University, Nanjing 210008, China

**Keywords:** robotic surgery, posterior approach, urinary continence, autologous vas deferens, clinical trial

## Abstract

**Highlights:**

**What are the main findings?**

- An autologous vas deferens (AVD) sling improved immediate (69.0% vs. 55.0%) and 1-month (81.0% vs. 67.0%) urinary continence rates after Retzius-sparing robot-assisted radical prostatectomy (RS-RARP) in high-risk patients. The between-group difference decreased after postoperative month 2.
- In an exploratory subgroup analysis, patients with a preoperative membranous urethral length (MUL) < 12 mm appeared to derive greater early continence benefits from the AVD sling. However, interaction analysis did not show a statistically significant differential treatment effect.

**What are the implications of the main findings?**

- The AVD sling may serve as a safe, feasible, and low-cost adjunct to RS-RARP for improving early urinary continence recovery during the first postoperative month, particularly in high-risk patients with factors associated with delayed recovery.
- The findings suggest that the AVD sling provides additional anatomical support to vesicourethral anastomosis and may help reduce the early burden of postoperative incontinence without adversely affecting perioperative or oncological outcomes.

**Abstract:**

**Objectives**: The objective of this study was to evaluate the efficacy and safety of an autologous vas deferens (AVD) sling for improving early urinary continence after Retzius-sparing robot-assisted radical prostatectomy (RS-RARP). **Methods**: In this single-center, prospective, randomized trial, 200 patients at high risk for postoperative urinary incontinence were randomized to undergo RS-RARP with or without AVD sling suspension. The primary endpoint was immediate urinary continence, defined as the use of 0–1 safety pads/day within 7 days after catheter removal. Secondary outcomes included continence recovery up to 3 months, EPIC-26 and IPSSs, and subgroup analysis according to the preoperative membranous urethral length (MUL). Multivariable logistic regression was performed to identify predictors of continence recovery. **Results**: The AVD sling group showed higher immediate (69.0% vs. 55.0%; *p* = 0.041) and 1-month continence rates (81.0% vs. 67.0%; *p* = 0.024) than the RS-RARP group. EPIC-26 scores and IPSSs also favored the AVD sling group during the early postoperative period (both *p* < 0.01). In exploratory subgroup analyses, patients with preoperative MUL < 12 mm appeared to derive greater early continence benefit from the sling procedure. The AVD sling remained independently associated with continence recovery at the immediate (adjusted OR 2.23, *p* = 0.014) and 1-month (adjusted OR 2.60, *p* = 0.008) assessments. Differences between groups decreased after postoperative month 2. The operative time was longer in the AVD sling group, whereas complication rates and short-term oncological outcomes were similar between groups. **Conclusions**: The AVD sling may improve early urinary continence recovery during the first postoperative month after RS-RARP, particularly in high-risk patients with shorter preoperative MUL.

## 1. Introduction

Prostate cancer (PC) is one of the most common malignancies among men worldwide and represents a major public health burden [1,2]. Robot-assisted radical prostatectomy (RARP) is widely used for the treatment of localized and locally advanced PC. However, despite technical advances in robotic surgery, postoperative urinary incontinence (PPI) remains a major functional complication and may substantially affect postoperative quality of life [3].

Retzius-sparing robot-assisted radical prostatectomy (RS-RARP), first described by Galfano et al. [4], preserves anterior anatomical support structures involved in urinary continence and has been associated with improved early continence recovery compared with conventional RARP. Previous studies and meta-analyses have reported favorable early continence outcomes following RS-RARP [5]. Nevertheless, early postoperative urinary incontinence remains common in patients with risk factors associated with delayed continence recovery, including advanced age, obesity, larger prostate volume (PV), shorter membranous urethral length (MUL), diabetes mellitus, prior transurethral resection of the prostate (TURP), and locally advanced disease [6,7,8,9,10,11,12].

Several reconstructive techniques have been developed to facilitate postoperative continence recovery, including posterior reconstruction and periurethral sling procedures [13,14]. Cestari et al. [15] described an autologous vas deferens (AVD) sling positioned beneath the vesicourethral anastomosis during RARP and reported encouraging early continence outcomes. However, evidence regarding the role of this technique in patients at elevated risk for delayed continence recovery remains limited, and prospective randomized data are lacking.

The present randomized controlled trial was therefore conducted to evaluate whether AVD sling suspension during RS-RARP could improve early postoperative urinary continence recovery in patients at high risk for postoperative incontinence.

## 2. Materials and Methods

### 2.1. Patients and Ethics

This study was designed as a prospective, single-center, parallel-group randomized controlled trial. We enrolled 200 patients with clinically localized prostate cancer who were at high risk for postoperative urinary incontinence. High-risk status was defined by the presence of at least one of the following predefined risk factors: age > 65 years, body mass index (BMI) ≥ 25 kg/m^2^, PV > 40 mL, preoperative MUL < 12 mm, CCI ≥ 2 [16], diabetes mellitus, TURP, or locally advanced disease (clinical stage ≥ T3) [12]. Exclusion criteria included prostate cancer invasion of the vas deferens or metastatic prostate cancer.

**Figure 1:**
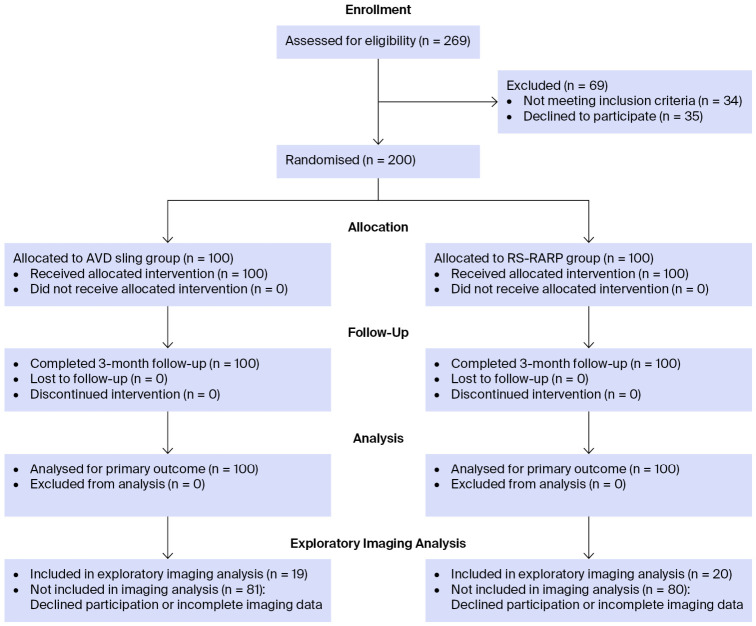
CONSORT 2025 flow diagram illustrating patient enrolment, randomization, allocation, follow-up, primary outcome analysis, and exploratory postoperative imaging analysis in patients undergoing RS-RARP with or without autologous vas deferens (AVD) sling suspension.

Eligible patients were randomly assigned in a 1:1 ratio to undergo RS-RARP with or without AVD sling suspension (Figure 1). The sequence was created independently by a statistician who was not involved in patient enrollment, surgical procedures, or postoperative outcome assessment. Randomization was performed using SAS version 9.4 (SAS Institute, Cary, NC, USA) with a predefined random seed and block randomization. Allocation concealment was maintained using sequentially numbered, sealed, opaque envelopes, which were opened only after induction of anesthesia in the operating room.

Given the nature of the surgical intervention, blinding of patients and surgeons was not feasible. Outcome assessors responsible for collecting data on pad usage and administering the EPIC-26 and IPSS questionnaires were blinded to treatment allocation throughout the follow-up.

All patients underwent RS-RARP. Patients in the AVD sling group underwent additional vesicourethral anastomotic suspension using an autologous vas deferens sling, as described in Section 2.2. Patients in the RS-RARP group underwent RS-RARP alone.

Written informed consent was obtained from all patients before enrollment. This study was approved by the Ethics Review Committee of Drum Tower Hospital affiliated with Nanjing University (2024-962-02) on 9 May 2025. The trial was registered with the Chinese Clinical Trial Registry (ChiCTR2500107175) on 5 August 2025.

### 2.2. Surgical Techniques

**Figure 2:**
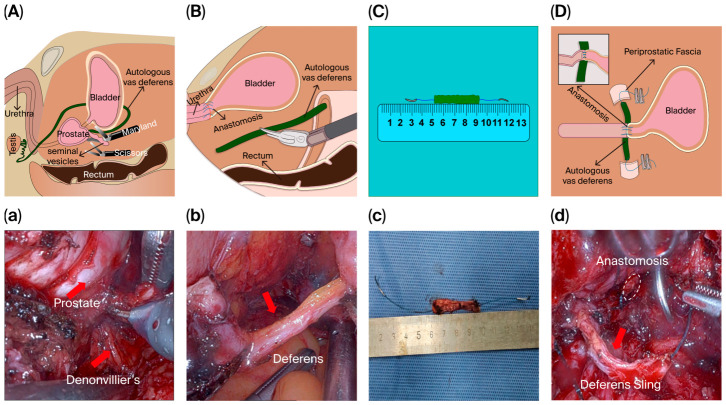
Surgical procedure of AVD sling suspension during RS-RARP. (**A**–**D**) Schematic illustrations of the surgical procedure. (**A**) Retzius-sparing dissection and mobilization of the vas deferens during RS-RARP. (**B**) Harvesting of the AVD and positioning beneath the vesicourethral anastomosis. (**C**) Extracorporeal preparation of the vas deferens sling using a double-armed suture. (**D**) Suspension of the vesicourethral anastomosis by fixation of the AVD sling to the surrounding periprostatic fascia. (**a**–**d**) Representative intraoperative images corresponding to the schematic illustrations. (**a**) Posterior dissection during RS-RARP with exposure of Denonvilliers’ fascia. (**b**) Intraoperative harvesting of the vas deferens. (**c**) Preparation of the autologous vas deferens sling. (**d**) Final placement of the AVD sling beneath the vesicourethral anastomosis after fixation.

The RS-RARP procedure was performed as previously described by Galfano et al. [4], with minor modifications. After robot docking, the peritoneum was incised at the Douglas pouch, and posterior dissection was performed along Denonvilliers’ fascia to the prostatic apex. The vas deferens and seminal vesicles were mobilized, followed by lateral dissection of the prostate. The bladder neck and urethra were transected, and vesicourethral anastomosis was subsequently performed (Figure 2A).

Pelvic lymph node dissection was performed according to oncological risk stratification and European Association of Urology guidelines. Extended pelvic lymph node dissection was performed in patients with intermediate-risk disease and an estimated risk of lymph node invasion > 5% according to the Briganti nomogram, as well as in patients with high-risk disease.

For the AVD sling procedure, the peritoneum was incised along the lateral umbilical ligament to expose the vas deferens. A segment approximately 4 cm in length was harvested (Figure 2B). Extracorporeal sling preparation was performed using a 10 cm 3-0 double-armed suture passed through the vas deferens (Figure 2C).

The vas deferens sling was positioned beneath the vesicourethral anastomosis and secured bilaterally to the surrounding prostatic fascia using the double-armed suture. The sling was tightened and fixed with hemostatic clips (Figure 2D). The surgical site was closed in layers, and a pelvic drain and transurethral catheter were placed at the end of the procedure.

### 2.3. Endpoints and Sample Size Calculation

Routine cystography was performed on postoperative day 14. Urinary catheters were removed only after confirmation of the absence of contrast extravasation. The primary endpoint was immediate urinary continence, defined as the use of 0–1 safety pads per day within 7 days after catheter removal. Urinary continence status was assessed during scheduled postoperative follow-up visits and telephone interviews. Pad usage was self-reported according to average daily pad use at each follow-up time point. Formal pad diaries and standardized pad weight testing were not routinely performed in this study.

Patient-reported outcomes were evaluated monthly using the International Prostate Symptom Score (IPSS) and the urinary domain of the Expanded Prostate Cancer Index Composite (EPIC-26) questionnaire. The EPIC-26 urinary domain was used to assess urinary continence and urinary symptom-related bother, whereas the IPSS was used to evaluate lower urinary tract symptoms. Postoperative complications occurring during the 3-month follow-up period were recorded and graded according to the Clavien–Dindo classification system.

The sample size was calculated based on the primary endpoint of immediate urinary continence. Assuming immediate continence rates of 40% in the RS-RARP group and 65% in the AVD sling group, with 90% power and a two-sided α level of 0.05, 99 patients per group were required. Sample size calculation was performed using PASS software version 15.0 (NCSS, LLC, Kaysville, UT, USA).

### 2.4. Statistical Analysis

The primary endpoint analysis was performed according to the intention-to-treat principle. The Pearson chi-square test or Fisher’s exact test was used for categorical variables. Continuous variables were compared using Student’s t-test or the Mann–Whitney U test, as appropriate. Non-normally distributed continuous variables were presented as the median and interquartile range (IQR), whereas normally distributed variables were presented as the mean ± standard deviation. Odds ratios (ORs) with 95% confidence intervals (CIs) were calculated.

Generalized estimating equations (GEEs) with an exchangeable correlation structure were used to evaluate longitudinal postoperative continence recovery across repeated follow-up assessments while accounting for within-subject correlation. Prespecified exploratory analyses of subgroups were performed according to preoperative MUL. Multivariable logistic regression analysis was performed to identify independent predictors of postoperative continence recovery. Variables included in the multivariable models were selected based on clinical relevance and previously reported associations with post-prostatectomy continence recovery. All selected variables were simultaneously entered into the regression models. Formal internal or external validation of the regression models was not performed, and the multivariable analyses were considered exploratory.

Statistical analyses were performed using SPSS version 21.0 (IBM SPSS, Chicago, IL, USA). A two-sided *p* < 0.05 was considered statistically significant.

## 3. Results

### 3.1. Baseline Demographics

A total of 200 eligible patients were randomized in a 1:1 ratio to the AVD sling group (*n* = 100) or the RS-RARP group (*n* = 100). No patients were lost to follow-up during the planned 3-month analysis period. Baseline demographic and clinical characteristics, including age, PV, preoperative MUL, comorbidity burden, PSA level, clinical stage, and smoking history, were comparable between the two groups (Table 1).

### 3.2. Primary Endpoint: Immediate Urinary Continence

In the intention-to-treat analysis (Table 2), all 200 patients were included in the primary endpoint assessment. Immediate urinary continence was achieved in 69.0% of patients in the AVD sling group compared with 55.0% in the RS-RARP group (*p* = 0.041; OR 1.82, 95% CI 1.02–3.25).

Exploratory subgroup analysis suggested a greater immediate continence benefit among patients with preoperative MUL < 12 mm (68.3% vs. 42.6%; OR 2.91, 95% CI 1.32–6.44; *p* = 0.008), although no significant interaction between treatment allocation and MUL subgroup status was observed (*p* for interaction = 0.143).

### 3.3. Secondary Endpoints: Continence Rates After Surgery and Urinary Function

All randomized patients completed the planned 3-month follow-up and were included in the present analysis. At 1 month postoperatively, continence rates remained higher in the AVD sling group than in the RS-RARP group (81.0% vs. 67.0%, *p* = 0.024). EPIC-26 urinary domain scores and IPSSs also favored the AVD sling group during the first postoperative month. No significant between-group differences in continence or patient-reported outcomes were observed thereafter.

Exploratory subgroup analysis suggested higher 1-month continence rates in patients with preoperative MUL < 12 mm undergoing AVD sling suspension than in those undergoing RS-RARP alone (81.7% vs. 57.4%; OR 3.30, 95% CI 1.38–7.90; *p* = 0.006). However, no significant treatment interaction across MUL subgroups was identified, and no significant between-group differences were observed beyond the first postoperative month.

Longitudinal generalized estimating equation analysis demonstrated a significant overall association between AVD sling placement and early postoperative continence recovery, with attenuation of the treatment effect over time.

### 3.4. Multivariable Analysis of Predictors for Postoperative Continence

**Figure 3:**
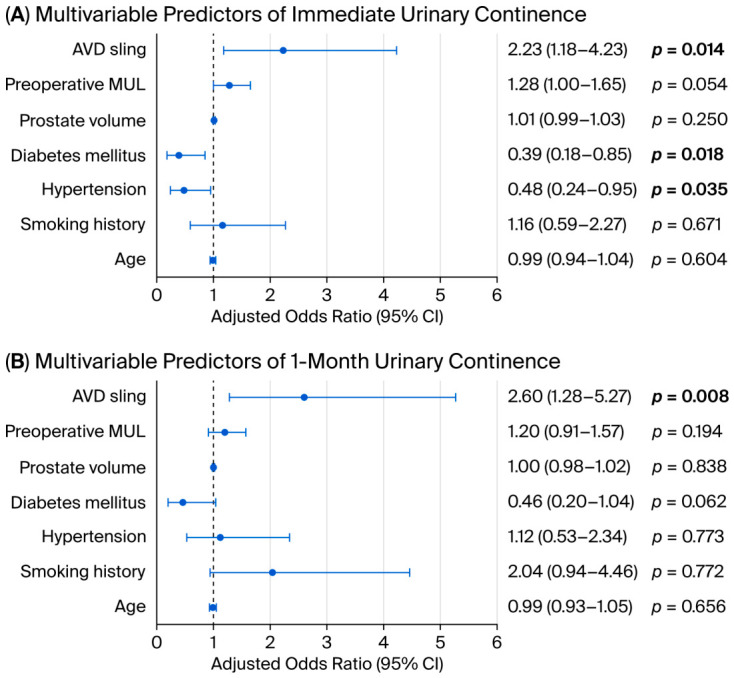
Forest plots showing selected clinically relevant predictors from multivariable logistic regression analyses of immediate (**A**) and 1-month (**B**) postoperative urinary continence recovery following RS-RARP. Adjusted odds ratios (ORs) and 95% confidence intervals (CIs) are presented. Statistically significant *p* values (<0.05) are shown in bold. The complete multivariable models including all covariates and later postoperative time points are provided in Supplementary Figure S1.

After multivariable adjustment, AVD sling placement remained independently associated with improved continence recovery in the immediate postoperative period and at 1 month. Diabetes mellitus and hypertension were associated with lower odds of early continence recovery, whereas larger prostate volume and smoking history were associated with continence status at 3 months postoperatively (Figure 3).

### 3.5. Intraoperative and Postoperative Outcomes and Pathological Data

Perioperative, pathological, and postoperative outcomes are summarized in Table 3. Estimated blood loss, hospital stay, pathological stage, ISUP grade, and positive surgical margin rates were comparable between the groups. Operative time was longer in the AVD sling group than in the RS-RARP group (142.5 vs. 135.0 min, *p* < 0.001). Two patients in the AVD sling group who received preoperative neoadjuvant therapy achieved complete pathological response.

No cases of acute urinary retention after catheter removal were observed in either group. Three Clavien–Dindo grade III complications occurred in the AVD sling group, including one urethral stricture and two pelvic abscesses, whereas two grade III pelvic abscesses occurred in the RS-RARP group. Complications were reported descriptively without definitive attribution to the sling intervention.

### 3.6. Exploratory Imaging Findings

An exploratory imaging analysis was performed in a subset of patients approximately 1 month after surgery. Postoperative imaging measurements were retrospectively evaluated by investigators blinded to treatment allocation. Nineteen patients in the AVD sling group and 20 patients in the RS-RARP group completed postoperative mpMRI, and all 39 patients underwent transrectal ultrasound examination.

**Figure 4:**
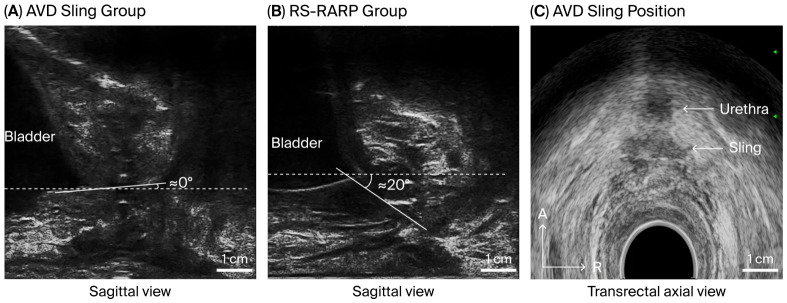
Representative postoperative imaging findings following RS-RARP with or without AVD sling suspension. (**A**,**B**) Comparison of the urethral inclination angle at resting status between the two groups. (**A**) In the AVD sling group, the proximal urethra demonstrates a nearly horizontal orientation. (**B**) In the RS-RARP group, the proximal urethra shows a more pronounced inclination angle. (**C**) A transrectal ultrasound image demonstrating the position of the autologous vas deferens sling beneath the vesicourethral anastomosis (the green triangles are part of the original ultrasound image generated by the ultrasound system).

**Figure 5:**
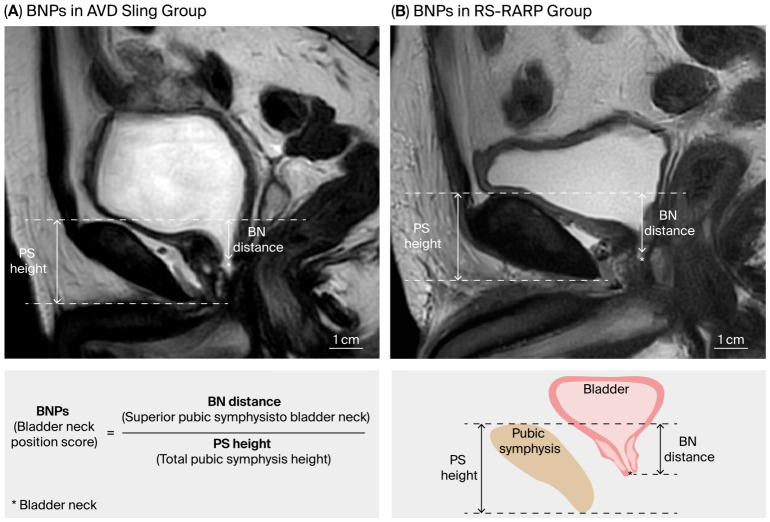
Measurement of bladder neck position score (BNPs) on postoperative sagittal T2-weighted MRI. BNPs was defined as the ratio of the distance from the superior pubic symphysis to the bladder neck (BN distance) to the total pubic symphysis height (PS height). (**A**) Representative image from the AVD sling group demonstrating a relatively higher postoperative bladder neck position. (**B**) Representative image from the RS-RARP group demonstrating a relatively lower postoperative bladder neck position. The schematic diagram illustrates the anatomical landmarks and measurement method used for BNPs calculation. * Indicates the bladder neck.

No significant between-group difference was observed in postoperative MUL (11.32 ± 1.09 mm vs. 11.03 ± 0.78 mm; *p* = 0.340). The AVD sling group demonstrated lower bladder neck-to-pubic symphysis ratios (BNPs) (0.55 vs. 0.64; *p* = 0.030) and smaller urethral inclination angles than the RS-RARP group (Figure 4 and Figure 5). Given the limited sample size, these imaging findings should be considered exploratory.

## 4. Discussion

Postoperative urinary incontinence remains a major functional complication after RARP. Disruption of the urethral sphincter and periurethral support structures is considered an important contributor to post-prostatectomy stress urinary incontinence [17]. Several reconstructive techniques, including posterior reconstruction and sling procedures, have been developed to facilitate continence recovery [18].

Recent studies have shown that RS-RARP is associated with earlier continence recovery than conventional RARP, without compromising perioperative or oncological outcomes [19,20]. This benefit is thought to be related to the preservation of anterior anatomical support structures and reduced disruption of the Retzius space.

Our randomized trial suggests that AVD sling suspension during RS-RARP may facilitate early postoperative continence recovery. To our knowledge, this is among the first prospective randomized studies evaluating this technique in an Asian population with an elevated risk of delayed postoperative continence recovery.

Our findings suggest that the benefit of the AVD sling is primarily limited to acceleration of continence recovery during the early postoperative period. Multivariable analyses showed an association between AVD sling placement and continence recovery in the immediate postoperative period and at 1 month, whereas this association was not observed at postoperative months 2 or 3. Different clinical factors were associated with continence outcomes at different postoperative time points. Diabetes mellitus and hypertension were associated with continence recovery in the immediate postoperative period, whereas prostate volume and smoking history were associated with continence status at 3 months. The association involving smoking history should be interpreted cautiously given the limited number of continence failure events at later postoperative time points.

Preoperative MUL is a well-established predictor of continence recovery after radical prostatectomy [21]. In our exploratory subgroup analysis, patients with MUL < 12 mm showed higher early continence rates in the AVD sling group than in the RS-RARP group. However, no significant interaction between treatment allocation and MUL subgroup status was observed. Therefore, these findings should be considered exploratory and hypothesis-generating.

In the multivariable analyses, preoperative MUL was not independently associated with early continence recovery after adjustment for surgical intervention and other clinical variables. One possible explanation is that the anatomical support provided by the sling may partially reduce the influence of shorter MUL on early postoperative continence recovery.

The benefit of the AVD sling appeared to be primarily limited to the early postoperative period. In our study, higher continence rates were observed during the immediate and first postoperative month, whereas no sustained difference was observed thereafter. Although the magnitude of benefit was limited to early recovery, postoperative urinary incontinence during this period may substantially affect patient quality of life, particularly in patients at high risk for delayed recovery. Therefore, acceleration of continence recovery may still provide short-term functional benefits during the early postoperative phase.

At the same time, the absence of a long-term functional advantage does not support routine universal implementation of this technique, and its use may be more appropriate in selected high-risk patients. These findings may warrant further investigation into anatomical factors related to continence recovery. In our exploratory imaging analysis, lower postoperative BNP values and smaller urethral inclination angles were observed in the AVD sling group, which may suggest a potential supportive effect of the sling on the vesicourethral complex [22,23]. However, these imaging findings remain exploratory and require validation in future studies with dedicated imaging assessment.

Our findings also provide additional context for the use of sling techniques during prostatectomy. Punnen et al. described a retropubic sling technique and suggested a potential role in facilitating continence recovery [24]. In our study, the sling was positioned beneath the vesicourethral anastomosis and fixed to the surrounding fascia within the Retzius-sparing approach, which may partially explain the early continence benefit observed.

In addition, the immediate continence rate in our control group was lower than those reported in some previous randomized studies [25,26,27], likely reflecting the inclusion of a higher-risk patient population.

Our study has several limitations. First, this was a single-surgeon, single-center study, which may limit the generalizability of the findings. Functional outcomes may have been influenced by the surgeon’s experience with RS-RARP and the AVD sling technique. Second, the study primarily focused on early continence recovery, and the follow-up duration was relatively limited. Consequently, long-term functional outcomes, including sexual function, broader quality-of-life measures, late postoperative complications, and long-term oncological safety, were not systematically assessed. Third, although postoperative outcome assessors were blinded to treatment allocation, blinding of surgeons and patients was not feasible because of the nature of the intervention. Continence outcomes also primarily relied on patient-reported pad usage. In addition, urinary continence was defined as the use of 0–1 safety pads per day, and objective pad weight testing was not routinely performed, which may limit comparison with studies using stricter continence definitions. Fourth, the exploratory imaging analysis was based on a relatively small subset of patients and was not powered to establish definitive mechanistic conclusions. Formal interobserver variability assessment for imaging measurements was also not systematically performed. Therefore, these imaging findings should be considered exploratory. Finally, the predefined high-risk cohort was clinically heterogeneous, and residual confounding may still have influenced the observed treatment effects. No formal adjustment for multiple comparisons was performed; therefore, findings from secondary endpoints and subgroup analyses should be interpreted cautiously.

Future multicenter studies with longer follow-up, blinded assessment, and collection of comprehensive longitudinal patient-reported outcomes are needed to further validate these findings.

## 5. Conclusions

AVD sling suspension during RS-RARP was associated with improved early postoperative continence recovery in patients at elevated risk for delayed continence recovery. Higher continence rates were observed immediately after catheter removal and during the first postoperative month, whereas no sustained between-group differences were observed thereafter. Exploratory subgroup analysis suggested a possible early benefit among patients with shorter preoperative MUL.

The observed benefit appeared to reflect acceleration of early continence recovery rather than durable long-term functional improvement. Further studies with larger sample sizes and longer follow-up are required to clarify the long-term clinical significance and reproducibility of this technique.

## Figures and Tables

**Table 1:** Baseline characteristics and predefined risk factors for postoperative urinary incontinence.

Variable	AVD Sling Group *n* = 100	RS-RARP Group *n* = 100	*p* Value
Predefined risk factors, *n* (%)			
Age > 65 years	84 (84.0)	86 (86.0)	0.692
BMI ≥ 25 kg/m^2^	40 (40.0)	42 (42.0)	0.774
Prostate volume > 40 mL	31 (31.0)	28 (28.0)	0.642
Preoperative MUL < 12 mm	60 (60.0)	47 (47.0)	0.065
CCI ≥ 2	32 (32.0)	36 (36.0)	0.550
Diabetes mellitus	28 (28.0)	25 (25.0)	0.631
Clinical stage ≥ T3	9 (9.0)	9 (9.0)	0.999
Prior TURP	7 (7.0)	10 (10.0)	0.447
Baseline characteristics			
Age, years	72.5 ± 7.2	71.1 ± 5.3	0.122
BMI, kg/m^2^	24.3 ± 3.0	24.5 ± 3.0	0.713
PSA, ng/mL	9.1 (5.0, 17.6)	7.5 (4.9, 12.6)	0.137
Prostate volume, mL	33.9 (23.8, 43.9)	31.3 (24.4, 42.7)	0.813
Preoperative MUL, mm	11.8 ± 1.3	12.0 ± 1.3	0.368
Hypertension, *n* (%)	63 (63.0)	62 (62.0)	0.884
Neoadjuvant endocrine therapy, *n* (%)	12 (12.0)	13 (13.0)	0.831
Biopsy ISUP grade, *n* (%)			
Grade 1	16 (16.0)	19 (19.0)	0.920
Grade 2	33 (33.0)	32 (32.0)	
Grade 3	27 (27.0)	23 (23.0)	
Grade 4	20 (20.0)	23 (23.0)	
Grade 5	4 (4.0)	3 (3.0)	
Clinical stage, *n* (%)			
cT1a–1c	3 (3.0)	2 (2.0)	0.298
cT2a	22 (22.0)	32 (32.0)	
cT2b	21 (21.0)	25 (25.0)	
cT2c	45 (45.0)	31 (31.0)	
cT3a–3b	9 (9.0)	9 (9.0)	
Smoking status, *n* (%)			
Never smoker	69 (69.0)	63 (63.0)	0.370
Current smoker	31 (31.0)	37 (37.0)	

Abbreviations: BMI, body mass index; CCI, Charlson comorbidity index; MUL, membranous urethral length; PSA, prostate-specific antigen; TURP, transurethral resection of the prostate. Data presentation: Continuous variables are presented as mean ± standard deviation or median (interquartile range), as appropriate. Categorical variables are presented as numbers (%).

**Table 2:** Postoperative urinary continence recovery and patient-reported outcomes within 3 months.

Outcome	AVD Sling Group (*n* = 100)	RS-RARP Group (*n* = 100)	*p* Value
Urinary continence recovery, *n* (%)			
Immediate postoperative period			
Continent (0–1 pad/day)	69 (69.0)	55 (55.0)	0.041
Postoperative month 1			
Continent (0–1 pad/day)	81 (81.0)	67 (67.0)	0.024
Postoperative month 2			
Continent (0–1 pad/day)	90 (90.0)	84 (84.0)	0.207
Postoperative month 3			
Continent (0–1 pad/day)	91 (91.0)	90 (90.0)	0.809
Patient-reported outcomes			
Epic-26 urinary domain score			
Postoperative month 1	74.5 (65.0, 85.0)	71.0 (52.0, 77.0)	0.002
Postoperative month 2	82.0 (71.5, 89.0)	77.5 (69.0, 88.0)	0.062
Postoperative month 3	88.0 (80.0, 92.0)	86.0 (77.0, 92.0)	0.535
IPSS			
Postoperative month 1	8.0 (6.0, 10.0)	9.00 (7.0, 11.0)	0.001
Postoperative month 2	7.0 (6.0, 8.0)	7.0 (7.0, 9.0)	0.059
Postoperative month 3	6.0 (5.0, 7.0)	7.0 (6.0, 8.0)	0.177

Abbreviations: EPIC-26, Expanded Prostate Cancer Index Composite-26; IPSS, International Prostate Symptom Score. Data presentation: Continuous variables are presented as median (interquartile range). Categorical variables are presented as numbers (%).

**Table 3:** Perioperative, pathological, and postoperative outcomes.

Variable	AVD Sling Group (*n* = 100)	RS-RARP Group (*n* = 100)	*p* Value
Perioperative outcomes			
Operative time, min	142.5 (135.0, 155.0)	135.0 (121.3, 148.8)	<0.001
Estimated blood loss, mL	100 (50, 100)	100 (50, 100)	0.916
Length of stay, days	6.5 (5.3, 7.0)	7.00 (6.00, 8.00)	0.541
Pathological outcomes			
Pathological T stage, *n* (%)			
pT0	2 (2.0)	0 (0)	0.562
pT2	58 (58.0)	54 (54.0)	
pT3a	34 (34.0)	38 (38.0)	
pT3b	6 (6.0)	8 (8.0)	
Pathological N stage, *n* (%)			
pNx	28 (28.0)	32 (32.0)	0.592
pN0	69 (69.0)	63 (63.0)	
pN1	3 (3.0)	5 (5.0)	
Pathological M stage, *n* (%)			
pMx	16 (16.0)	9 (9.0)	0.134
pM0	84 (84.0)	91 (91.0)	
Pathological ISUP grade, *n* (%)			
Grade 1	10 (10.0)	10 (10.0)	0.928
Grade 2	39 (39.0)	44 (44.0)	
Grade 3	38 (38.0)	32 (32.0)	
Grade 4	9 (9.0)	9 (9.0)	
Grade 5	4 (4.0)	5 (5.0)	
Positive surgical margins, *n* (%)	30 (30.0)	35 (35.0)	0.450
Clavien–Dindo grade III complications, *n* (%)			
Urethral stricture	1 (1.0)	0 (0.0)	0.999
Pelvic abscess	2 (2.0)	2 (2.0)	

Abbreviation: ISUP, International Society of Urological Pathology. Data presentation: Continuous variables are presented as median (interquartile range). Categorical variables are presented as number (%).

## Data Availability

The data presented in the study are available on request from the corresponding author due to ethical and privacy restrictions. This study was approved by the Ethics Review Committee of Drum Tower Hospital Affiliated to Nanjing University (approval No. 2024-962-02), and the informed consent obtained from participants did not include provisions for public date deposition. Requests for de-identified participant date should be directed to the corresponding author (Dr. Qing Zhang, drzhangq@nju.edu.cn) and will be considered on a case-by-case basis.

## Supplementary Materials

The following supporting information can be downloaded at: https://www.mdpi.com/article/10.3390/healthcare14142090/s1, Figure S1: Multivariable logistic regression analyses of predictors associated with postoperative urinary continence recovery at different postoperative time points.
