# Supplementary material for: Autologous Vas Deferens Sling for Early Urinary Continence During Retzius-Sparing Robot-Assisted Radical Prostatectomy: A Randomized Controlled Clinical Trial
Source: Healthcare (Basel). 2026 Jul 13;14(14):2090. doi: 10.3390/healthcare14142090 (PMC13409912; doi:10.3390/healthcare14142090)
Supplement: Supplementary file 1 [file healthcare-14-02090-s001.zip › healthcare-4257309-supplementary.pdf]

Predictor	Immediate continence		1 month after surgery		2 month after surgery		3 month after surgery	
	Adjusted OR (95%CI)	P value	Adjusted OR (95%CI)	P value	Adjusted OR (95%CI)	P value	Adjusted OR (95%CI)	P value
**Treatment group**								
AVD sling	2.23 (1.18−4.23)	**0.014**	2.60 (1.28−5.27)	**0.008**	2.10 (0.83−5.29)	0.117	1.24 (0.46−3.89)	0.586
**Anatomical & Tumor Factors**								
Preoperative MUL (mm)	1.28 (1.00−1.65)	0.054	1.20 (0.91−1.57)	0.194	1.11 (0.78−1.56)	0.564	0.92 (0.62−1.36)	0.663
Prostate Volume (ml)	1.01 (0.99−1.03)	0.250	1.00 (0.98−1.02)	0.838	1.02 (0.98−1.06)	0.351	1.09 (1.02−1.16)	**0.006**
PSA (per ng/ml)	0.99 (0.96−1.03)	0.652	0.99 (0.95−1.03)	0.678	0.99 (0.94−1.04)	0.734	0.96 (0.91−1.01)	0.136
**Comorbidities & Habits**								
Diabetes Mellitus	0.39 (0.18−0.85)	**0.018**	0.46 (0.20−1.04)	0.062	0.51 (0.18−1.42)	0.196	0.53 (0.14−1.95)	0.336
Hypertension	0.48 (0.24−0.95)	**0.035**	1.12 (0.53−2.34)	0.773	1.28 (0.47−3.46)	0.626	1.29 (0.39−4.28)	0.680
Smoking History	1.16 (0.59−2.27)	0.671	2.04 (0.94−4.46)	0.072	1.97 (0.65−5.92)	0.230	7.05 (1.32−37.58)	**0.022**
Charlson Index (point)	1.09 (0.82−1.44)	0.563	0.92 (0.68−1.25)	0.608	0.78 (0.54−1.12)	0.175	0.97 (0.62−1.53)	0.898
**Other Clinical Factors**								
Age (years)	0.99 (0.94−1.04)	0.603	0.99 (0.93−1.05)	0.656	0.97 (0.90−1.05)	0.418	0.96 (0.87−1.05)	0.337
BMI (kg/m²)	0.99 (0.89−1.11)	0.865	0.96 (0.85−1.09)	0.556	1.01 (0.86−1.18)	0.896	0.91 (0.76−1.10)	0.321
Prior Urethral Surgery	1.07 (0.36−3.22)	0.902	0.90 (0.27−2.95)	0.861	0.40 (0.10−1.58)	0.190	0.43 (0.08−2.16)	0.303
Neoadjuvant Therapy	1.31 (0.47−3.67)	0.612	1.29 (0.40−4.17)	0.669	1.98 (0.35−11.34)	0.442	6.80 (0.58−79.64)	0.127

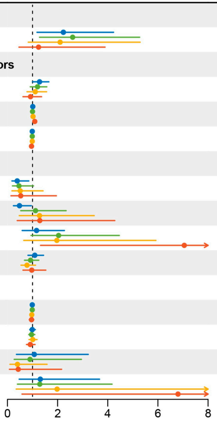

Figure S1. Multivariable logistic regression analyses of predictors associated with postoperative urinary continence recovery at different postoperative time points. Forest plot showing adjusted odds ratios (ORs) and 95% confidence intervals (CIs) from multivariable logistic regression analyses evaluating clinical predictors of urinary continence recovery immediately after catheter removal and at 1, 2, and 3 months after surgery. Variables included treatment allocation, anatomical and tumor-related factors, comorbidities, lifestyle factors, and other clinical characteristics. The AVD sling group demonstrated a significant association with improved immediate continence recovery and 1-month continence recovery, whereas no significant association was observed at postoperative months 2 or 3. Odds ratios greater than 1 indicate a positive association with continence recovery. Statistically significant associations are shown in bold.
